# Effectiveness- and cost effectiveness of a structured method for systematic and integrated occupational safety and health and patient safety management systems (SIOHPS) – a study protocol for a pragmatic stepped wedge cluster randomised controlled trial

**DOI:** 10.1186/s12913-025-13537-4

**Published:** 2025-10-22

**Authors:** Malin Lohela-Karlsson, Ann-Sofie Ersson, Therese Hellman, Emelie Condén Mellgren, Gunnar Bergström, Petronella Bjurling-Sjöberg, Robert Sarkadi Kristiansson, Camilla Göras

**Affiliations:** 1 Centre for Clinical Research, Region Västmanland, Västmanland Hospital Västerås, Uppsala University, Västerås, Sweden; 2 Department of Public Health and Caring Sciences, Uppsala University, Uppsala, Sweden; 3 School of Health, Care and Social Welfare, Mälardalen University, Västerås, Sweden; 4 Department of Medical Sciences, Uppsala University, Uppsala, Sweden; 5 Department of Occupational and Environmental Medicine, Uppsala University Hospital, Uppsala, Sweden; 6 Unit of Intervention and Implementation Research for Worker Health, Karolinska Institutet, Stockholm, 17177 Sweden; 7 Department of Occupational Health, Psychology and Sports Sciences, Faculty of Health and Occupational Studies, University of Gävle, Gävle, Sweden; 8 Centre for Clinical Research Sörmland, Uppsala University, Eskilstuna, Sweden; 9 Department of Caring Sciences, Faculty of Health and Occupational Sciences, University of Gävle, Gävle, Sweden; 10 School of Health and Welfare, Department of Caring Sciences, Dalarna University, Falun, Sweden

**Keywords:** Safety culture, Work environment, Patient safety, Healthcare organizations, Psychological safety, Intervention, Safety I, Safety II, Occupational safety and health

## Abstract

**Background:**

Integrated occupational safety and health and patient safety management are essential for addressing the challenges faced by healthcare services today. Developing and evaluating tools that support this work is crucial. This project aims to assess the effectiveness of a structured method for systematic and integrated occupational safety and health and patient safety management systems (SIOHPS). Additionally, the project includes embedded economic and process evaluation. This article presents the overall design of the SIOHPS-project, with a specific focus on the design and evaluation of the (cost-)effectiveness study.

**Methods:**

The project is guided by the Medical Research Council (MRC) framework for complex interventions and is coproduced with key stakeholders. The intervention is designed to support systematic occupational health and patient safety management systems, incorporating both Safety I and Safety II perspectives. It is grounded in safety culture theory and knowledge about team debriefing for learning. The intervention consists of several core components, including targeted education, end-of-shift team debriefings, and support for systematic management. The intervention is supported by a digital tool. A program theory guides the evaluation. A pragmatic stepped-wedge cluster-controlled design (p-SWD) is used, with hospital healthcare units as clusters. The p-SWD includes three steps, with at least four clusters transitioning from the control to the intervention group at each step. A minimum of twelve healthcare units from two different regions in Sweden will participate. The intervention effect will be evaluated using sick leave and quality of care as primary outcomes. Secondary outcomes include safety climate, work environment factors, healthcare worker health, performance, patient safety and quality of nursing care. Primary and secondary analyses are conducted based on intention-to-treat approach. Cost-effectiveness will be assessed using cost-benefit and cost-consequence analyses.

**Discussion:**

The need of methods that integrate systematic occupational safety and health and patient safety management has been emphasized by different stakeholders worldwide. The SIOHPS study has strong potential for nationwide implementation in Sweden to help healthcare organizations address current challenges. Additionally, the project will contribute to existing safety culture theory by exploring the integration of these domains.

**Trial registration:**

ClinicalTrials.gov Identifier: NCT06398860. Registration date: 2024-04-30.

**Supplementary Information:**

The online version contains supplementary material available at 10.1186/s12913-025-13537-4.

## Background

To meet the demands of a complex adaptive system such as healthcare, providing high quality care and ensuring sustainability through a healthy workforce, a higher degree of integration between occupational safety and health (OSH) and patient safety (PS) is proposed [1–4]. Due to the association between these fields risk factors in one area likely have consequences on the other and, if handled in an integrated manner, adverse consequences in both areas could be prevented simultaneously. Besides potentially positive effects on both healthcare workers (HCW) and quality of care, common methods, tools and actions instead of separate ones could contribute to more effective use of resources. However, there is a lack of research-based methods that are evaluated based on the impact of integrated systematic OSH and PS management on safety culture, HCW health and quality of care. A systematic review [5] concludes that interventions to improve patient safety culture (PS culture) in hospital settings may benefit HCW working conditions and health. The interventions were, however, not designed to support systematic OSH management or to improve OSH culture. In a recent scoping review [6] the authors aimed to identify interventions developed to simultaneously strengthen both OSH culture and PS culture in healthcare settings. No studies were found that captured an integrated perspective, indicating a need for developing and evaluating such methods.

One way of understanding how to create safer healthcare for both patients and HCW is through the lens of safety culture as described in the Safer Culture Framework [7]. Developed from a comprehensive literature review, this theoretical model describes how collective attitudes, values and behaviors that prioritize safety are influenced by enabling factors and enacting behaviors. Enabling factors at organizational, group and individual levels, for example management prioritization of safety, cohesion, and individual commitment, and enacting behaviors, for example teamwork and incident reporting, interact at different organizational levels in healthcare situations to improve the safety culture and create safety outcomes. One of the most critical factor is that managers prioritize safety [8], both by creating opportunities for the HCW to engage in safety behaviors and to keep them motivated by addressing identified risks. An enabling factor of high interest in safety culture and for team performance, is psychological safety, a state where it is safe for team-members to voice concerns or admit mistakes without taking interpersonal risks [9]. Psychological safety is built in the work environment through interactions within the team and are just as important for the wellbeing of the HCW as for the safety of the patients [10]. To date, there is extensive research on the importance of psychological safety in a variety of settings, and it mediates or moderates outcomes such as learning and performance [11]. This suggests that psychological safety is an important mechanism of impact to assess and consider in studies.

Team based debriefing methods have been found effective for learning and performance [12, 13] and can be conducted with limited resources, which is important for adherence and sustainability [14]. Available methods are many and vary, for example after action reviews and safety huddle, and can, if used properly, be effective tool to strengthen both PS and HCW wellbeing [12, 13, 15, 16]. The core components of these methods, such as communication, information exchange and incident reporting, are related to the enacting behaviors in the Safer Culture Framework [7]. Team debriefing fits into the healthcare context and can be conducted regularly to identify, act on, and learn from current issues.

To understand and cope with safety challenges in a complex adaptive system, it is important not just to manage risks and adverse events, i.e. the traditional Safety I-approach, but also to identify strengthening factors, the Safety II-approach [17, 18]. To reach resilient healthcare and high safety for both PS and OSH, a comprehensive understanding of the system as a whole, including its various components and interactions [19], and learning from both safety approaches is essential.

For a long-lasting result the methods need to engage the enabling factors at several system levels, consist of core components related to the enacting behaviors and make sure that these are targeted simultaneously. To work integrated with effective results, i.e., creating an environment that is safe for both HCWs and their patients, leading to enhanced HCW health and quality of care, there is a need to develop and evaluate methods including both approaches to safety and targeting factors at different organisational levels. Moreover, it is essential to evaluate the process of developing, implementing, and utilizing a new tool and method. Therefore, a project was launched to develop and evaluate a structured method for systematic and integrated OSH and PS management systems (SIOHPS). This paper describes the overall design of the project and provides a protocol for a study aiming to evaluate the effectiveness and cost-effectiveness of the SIOHPS method.

## Research questions

Can the use of a structured method for systematic and integrated occupational safety and health and patient safety management systems lead to:

Primary outcomes:

1. Reduced sick leave?
2. Improved quality of care?

Secondary outcomes:

1. Improved working conditions?
2. Enhanced safety culture?
3. Improved HCW health?
4. Improved performance?
5. Improved patient safety?
6. Improved quality of nursing care?

## Methods/design

### Study design of the SIOHPS-project

The SIOHPS-project is a multidisciplinary complex intervention study conducted in collaboration between researchers from several disciplinary fields with complementary clinical experiences and academic experience. The project is coproduced with key stakeholders, including managers, HCW involved in PS-tasks, safety representatives and central functions with responsibility for developing PS and OSH management structures. The project is guided by the Medical Research Council (MRC) framework for complex interventions [18] and consists of two main components: a development phase and an evaluation phase. Project preparation started in 2022. In the development phase, which was conducted during 2023–2024, a program theory, the intervention, and the evaluation design were refined according to the process described by O´Cathain et al. [20].

**Fig. 1:**
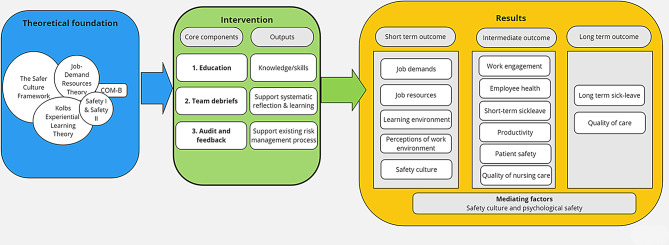
The programme theory of the SIOHPS intervention, illustrating the theoretical foundation, the core components and expected outputs of the intervention, and expected short, intermediate and long-term results as well as potential mediating factors

The SIOHPS intervention is a method for systematic integration of OSH and PS management, developed for healthcare organizations. The method includes three core components supported by a digital tool that, based on the program theory, are expected to produce the desired intervention result (Fig. 1).

In the evaluation phase (July 2024 to April 2026) the SIOHPS method will be tested using a pragmatic stepped wedge cluster-controlled design (p-SWD). An effectiveness-implementation hybrid design Type I [21] is employed, primarily aiming to determine effectiveness and cost-effectiveness of the intervention and secondarily, in a process evaluation, to describe what worked or did not work, adaptations and required support when implementing the complex intervention.

**Fig. 2:**
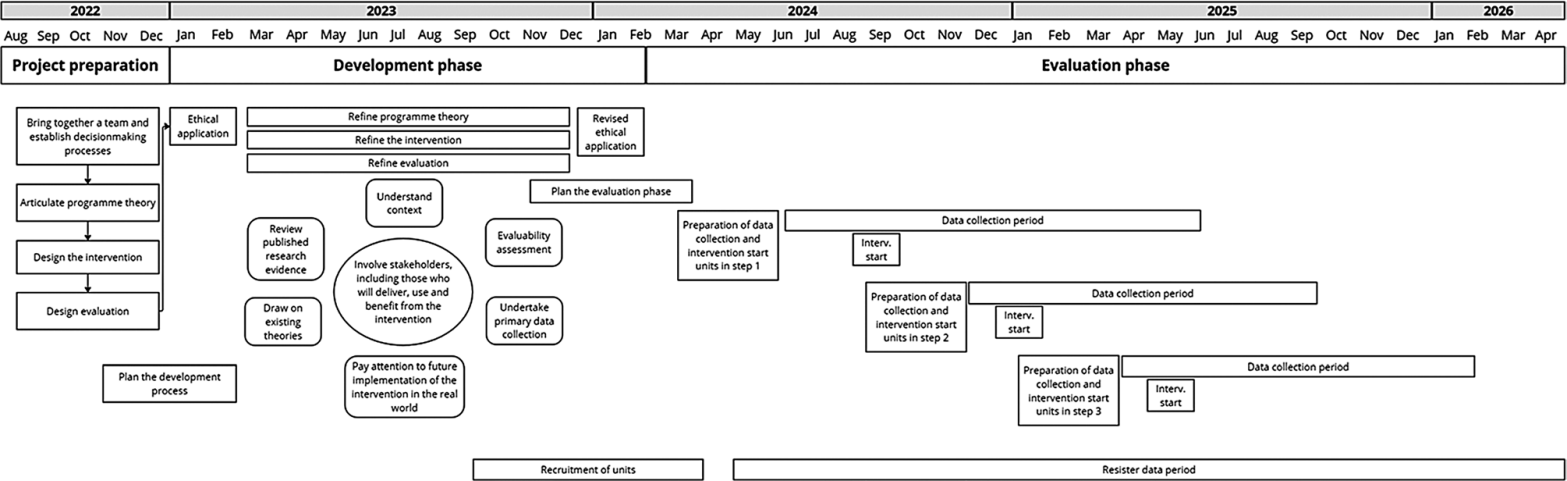
Overview of the different phases in the research project and activities

An overview and timeline of the project are presented in Fig. 2.

Study completion and reporting will be conducted in accordance with the Consolidated Standards of Reporting Trials (CONSORT) [22] and the Template for Intervention Description and Replication (TIDieR) [23]. The study is registered in Clinical Trials.gov (ID number NCT06398860) and is approved by the Swedish Ethics Review Authority (ID number 2023-02402-01 and 2024-01407-02).

### Program theory

The program theory of the SIOHPS-intervention is based on five theoretical foundations; the Safer Culture Framework [7], the Job Demands Resources (JDR) Theory [24], The Safety I and II approach [25], Kolb’s Experiential Learning Theory [26] and COM-B Model for Behaviour Change [27]. The Safer Culture Framework [7] provides overall guidance for the intervention together with the JDR theory [24]. The Safer Culture Framework underpins that interventions should adopt an integrated approach that supports both enabling factors and enacting behaviors and targets organisational, group and individual levels, which has been considered in the development of the SIOHPS method.

The JDR theory incorporates safety culture with working conditions and their consequences for both the individual employee and the organization. It further explains how working conditions influence HCW´s psychological well-being and how demands can cause stress that can be mitigated with autonomy and control over the work situation. The SIOHPS method is assumed to facilitate HCW participation and influence at work, which is expected to increase perceived control over the work situation. The theory provides additional theoretical foundation for the intervention and expected outputs and results.

The Safety I and II approach [25], Kolb’s Experiential Learning Theory [26] and the COM-B Model for Behaviour Change [27] provide complementary perspectives and are primarily used in the design of the different intervention core components. The Safety I and II approach [25] suggests that PS is promoted by understanding incidents, risks, no-harm incidents, and adverse events as well as successes. In the present project it is also intended to strengthen HCW confidence and wellbeing, which is important for learning and positive development of the safety culture. Kolb’s Learning Theory is the underlying base of team debriefing that engages HCWs in reflective practices about their work experiences [12, 28]. Kolb´s Learning Theory highlights how experiences, including thoughts and emotions, create a continuous process of learning that is key for development. The evidence of how team debriefings are best designed to achieve desirable effects is considered in the intervention.

To facilitate sustainability in learned behavior, the intervention also builds on the COM-B model [27], an acronym that stands for capability, opportunity, motivation and behavior. The first three components need to be considered to create behavior change, in this case, maintaining the regularly team debriefings over time at the units.

Through the core components, the intervention is expected to enhance HCW participation and learning, and to create conditions that improve the short-term outcomes, i.e. job demands, job resources, learning environment, perceptions of the work environment and safety culture. In turn, this is expected to have a positive effect on the intermediate outcomes, i.e. work engagement [29], HCW health [30, 31], productivity, patient safety [32–34] and perceived quality of care [34], which will positively impact long term sick-leave rates and quality of care [32, 35]. The effects are suggested to be mediated by safety culture and psychological safety [11], see Fig. 1.

### Intervention

The SIOHPS method consists of three core components: education, structured team debriefing, and audit and feedback. Before starting the complex intervention in a setting, the managers assign a couple of HCW as internal facilitators to support the activities in the team debriefing [36]. The facilitators should preferably be of different professional backgrounds, representing the composition of HCW at the unit.

To facilitate the utilization of SIOHPS and adherence to the method, a novel digital tool is provided, developed by the researchers in collaboration with key stakeholders. The digital tool includes reflection questions to support the team debriefings and visualized summaries. All HCWs can use the tool in the team debriefings and view a summary of the monthly assessments, events, lessons learned, and improvement suggestions. Managers and support functions also have access to a statistics view with various selection options, to be used for audit and feedback.

### Component 1 -Education

The education aims to increase the knowledge foundation and provide practical skills to utilize the SIOHPS method and is designed to target the needs of different system levels.

First, a two-hour interactive education is conducted for management and the support functions; those involved in patient safety work, the safety representatives of the work environment (hereafter just called safety representative) and the internal facilitators. The education includes information on (1) OSH, PS, concepts, and systematic management systems, (2) safety culture and psychological safety, (3) demonstration of the content of the intervention including the team debriefing method with the supporting digital tool and (4) case discussions with a special focus on managers´ responsibility in audit and feedback. A checklist and written information that addresses practical issues and advice to support the team debriefing are provided. Second, close to intervention start, all HCWs at each unit participate in a short version of the above-described education (approximately 45 min) with similar content and the possibility to ask questions.

The education is provided by two of the researchers; A-SE, a specialist psychologist in work and organizational psychology, and CG, a specialist registered nurse and PS researcher. Education for managers and support functions is conducted digitally and delivered jointly by A-SE and CG. Education for the HCWs are held physically at the units by either ASE or CG. For non-present and thereafter newly hired HCWs, a digital version of the education is offered, and the shorter version is also available in the digital tool. Guidance in initiating the team debriefings is provided to the internal facilitators.

### Component 2 -Team debriefing

The second core component is end-of-shift team debriefings, utilizing the digital tool available to all HCW at the unit. The structure is standardized to support the identification of situations related to both OSH and PS and consists of three steps. First, HCW *reflect* on how their work shift was, including things that went well, less well or were difficult. In the second step HCW *assess* the work environment and PS during the work shift using a traffic light system (green, yellow, red). These assessments are made separately but are designed jointly to remind that these are often ”two sides of the same coin”. The color green represents a work shift where the work environment/PS was good, a yellow assessment represents a work shift where risks or nearby-accidents (work environment) or no-harm incidents (PS) were identified, and red represents a work shift with an identified work injury or a patient affected by injury, i.e. adverse event [37].

In the third step, HCW focus on *learning* by reflecting on what they learned during the work shift, including things that went well and things that could be improved. The HCWs are encouraged to share concrete examples, to give each other support and feedback, and to make notes in the digital tool. However, they are instructed that learning in respectful dialogues is more important than the documentation or to performing many “green” assessments. The debriefing time is limited, approximately 10 min, but extension is encouraged if needed, for example after stressful events. Additionally, the HCWs are instructed that they don’t have to solve all identified issues, but rather just make a note and inform managers for later discussions. If the work environment or PS is assessed as yellow or red, the ordinary incident reporting system is used according to current routines.

The team debriefs should be conducted at least once every 24 h but are encouraged to be conducted after each work shift. The units can adapt the debriefings based on the requirements in the setting and conduct the sessions in smaller parallel groups or with all HCW together. The team debriefings are designed to enable the HCWs to autonomously conduct the sessions. The internal facilitator supports the HCWs mainly at the start of the implementation and helps the managers to maintain adherence and sustainability.

The daily assessments with traffic light ratings are visualized per calendar monthly in a circle and are encouraged to be shown on a screen at the unit to monitor daily work.

### Component 3 -Audit and feedback

The third component includes audit and feedback, based on information identified in the team debriefings. This component targets management and should be conducted in collaboration with the support functions, i.e. those involved in patient safety work, the safety representatives and the internal facilitators, according to ordinary routines in the organization. They are recommended to use the daily ratings and analyze for potential patterns, search for root causes and take measures considering both OSH and PS from an integrated perspective. Managers are responsible for reviewing, discussing and giving feedback to the HCWs at least monthly about the collected data or the results, preferably at a unit meeting where everyone can participate. Participation of HCWs in the different steps of this component is encouraged.

### Study design

**Fig. 3:**
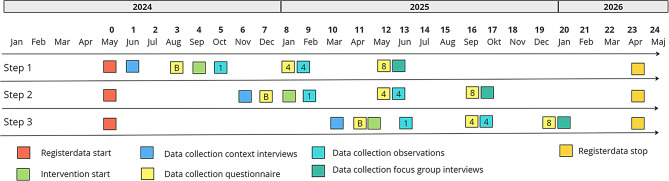
Time schedule of enrolment in the intervention and the different data collections that are carried out in the evaluation phase of the research project. The number in the different boxes represents the time (month) from intervention start when the data collection is carried out. “B"= Baseline, 1 = 1 month, 4 = 4 months, and 8 = 8 months

The effectiveness study is designed as a pragmatic stepped wedge cluster randomized controlled design (p-SWD) [38], with work units as clusters. A pragmatic (randomised) controlled trial aims to evaluate the effectiveness of complex interventions in real world settings, i.e. to investigate if the intervention works under usual conditions, and is designed to be conducted within the ordinary system [39]. Additionally, a broad use of eligibility criteria and minimal exclusion criteria is common. The stepped wedge design is suitable in intervention studies where all clusters could benefit from the intervention [40]. The SIOPHS-study is designed to consist of three steps, with at least four clusters crossing over from the control to the intervention group at each step. The study begins with a phase in which all clusters serve as controls. Subsequently, they transition to the intervention group in multiple steps. The transition to each new step occurs every four months, see Fig. 3. The intervention is utilized by participating clusters for eight months, but clusters in the initial steps are encouraged to continue using the intervention until the clusters in the last step have completed their intervention period.

### Procedure

The study is carried out in hospitals in two different Swedish regions. The recruitment of hospital units was conducted from September 2023 to March 2024. Information about the project was distributed to central PS representatives, HR-representatives and work environment specialists. Subsequently, further contact to anchor the project was established within the organization. First, through senior management and after informed consent, contact was established with first line managers. Information about the study and what participation entails was sent to managers by e-mail and followed up with information meetings. Recruitment of units continued until the required number was reached. Afterwards, the units were clustered and randomized to the different steps.

Close to when the unit transitions from the control group to the intervention group, the researchers provide further study information at workplace meetings with the opportunity for clarifying questions from HCWs. Participation in the interventions is encouraged regardless of the individual’s participation in the study, which is voluntary. Invitations to participate in the study are sent to the HCWs via their job e-mail addresses. Informed consent for study participation and access to sick-leave register data is collected when the baseline questionnaire is sent out. Reminders are sent out to encourage study participation and increase response rates. An open cohort design is used, meaning that new HWCs will be allowed to enter the study throughout the project period. They will provide informed consent as part of their first questionnaire regardless of when they are included in the study.

The time schedule of intervention enrollment and the different data collections are presented in Fig. 3. Register data and questionnaire data are used for the effectiveness evaluation. Parts of the questionnaires, together with individual interviews, observations, documentation related to the intervention components collected from the entire study period and focus group interviews are used in the process evaluation.

The research team maintains contact with managers and internal facilitators at participating units throughout the project period to strengthen intervention adherence. The research team meets weekly during the evaluation phase to monitor the project progress and to ensure that the data collection is conducted in accordance with the study protocol. Managers receive a workplace report with summative results from the questionnaires after the first and last data collection to stimulate a high response rate. Additionally, gift cards are raffled among responders after each data collection phase.

### Participants

Inclusion and exclusion criteria are applied at two levels. First, inclusion and exclusion criteria at the unit level are applied. To be eligible for the study, units need to provide round-the-clock care. Units with plans to implement any other OSH and/or PS improvement work during the project period, and units providing pediatric and psychiatric care are not eligible to participate in the study. The latter criteria are due to difficulties in evaluating the effect on decided outcome measures related to quality of care. Second, inclusion criteria are applied at the individual level. To be included in the study, HCWs must be employed at least 50% of full-time work at the unit. This is to ensure that they can participate in the intervention on a regular basis. All professions are encouraged to participate in the intervention, whether they fulfill the inclusion criteria or not, as teamwork is fundamental in safety culture.

### Sample size

The sample size was decided a priori using a power calculation tool; The Shiny CRT Calculator: Power and Sample size for Cluster Randomised Trials [41]. The effect was estimated at the HCW level (intervention effect) for the primary outcome of sick leave based on the following parameters: Power 80%, *p* < 0.05, clinically relevant change in the primary outcome of 30%, ICC = 0.01, number of “steps” = three, and an equal number of clusters switching to the intervention group on each occasion. Each cluster was assumed to consist of an average of 30 HCW. According to the calculation, a minimum of twelve units and 360 HCW needed to be included in the study to have enough statistical power for further analysis. This result is conservative enough to detect the lowest expected intervention effect. We regarded this sample size as the minimum number of units and HCW to include, with a primary focus on reaching the required number of units in the study as the number of HWCs varies between units.

### Randomization

A pragmatic randomization [42] was performed at the cluster level, where units were randomized to one of the three steps. Consideration was given to geographically neighboring clusters, collaboration between managers, and the number of HCW at the unit, when deciding cluster allocation for each step, aiming to minimize the risk of contamination and to ensure an even number of clusters and HCW in each step. Potential requests from managers, with strong arguments, were considered in the allocation procedure after discussions within the research team. For example, larger organizational changes that might affect their ability to start the intervention at a certain time point could result in their need to withdraw from the study. Since the project is designed with a pragmatic stepped wedge cluster randomized controlled design where the intervention is designed to work under usual conditions within the ordinary system [39], it is important to also consider and handle these prerequisites in the design of the study. The allocation sequence was generated by the research team at a joint meeting. First, any prerequisites were brought up and discussed, such as final number of units and the number of HCW at each unit, collaboration, and potential requests. Second, units with a risk of contamination were bundled to be randomized together. Units with special requests, approved by the research team, were assigned to the a priori step or restricted to two out of three steps in the randomization procedure. Third, a computer-generated random number was used for the allocation sequence in which each unit was assigned a number. There was constant reconciliation in the research team after each randomization sequence to ensure that the result across the three steps consisted of an even number of units and HCW.

### Blinding

As the intervention consists of active ingredients and involves HCWs at the workplaces, neither the manager nor the HCWs at the participating workplaces are blinded after assignment to the intervention. An unblinded data manager handles the raw data when exporting the data from organizational registers and the system used to collect the questionnaires. The data manager is responsible for merging the data files before handing a processed data file to a data analyst. The data analyst for the primary outcomes is blinded regarding the intervention allocation, i.e., to which step each cluster is allocated.

### Data collection procedure and outcomes

To evaluate the effectiveness of the intervention, data will be collected using register data and online-based self-reported questionnaires, see Table 1 for an overview of measures and outcomes.

**Table 1:** Overview of assessments and outcomes at each study time point

Outcome	Baseline	4-months follow-up	8 months follow-up	12 months follow-up
*Participant characteristics*				
Sex, age, educational level, occupation, years at current workplace, employment status, working hours, extent of overtime	X			
**Primary outcome**				
***Register data***				
Sick leave, total number of days	X	X	X	X
Quality of care - readmission rate within 30-days of discharge	X	X	X	X
**Secondary outcomes**				
***Self-reported measures***				
*Work environment-related aspects*				
*Job demands*				
Quantitative demands (COPSOQ)	X	X	X	
Work pace (COPSOQ)	X	X	X	
Emotional demands (COPSOQ)	X	X	X	
*Job resources*				
Local leadership (SCORE)	X	X	X	
Perception of management (SAQ)	X	X	X	
Teamwork climate (SAQ)	X	X	X	
Horizontal trust (COPSOQ)	X	X	X	
Meaningful work (COPSOQ)	X	X	X	
Influence at work (SAQ)	X	X	X	
Learning environment (SCORE)	X	X	X	
Perceptions of the work environment (RN4CAST)	X	X	X	
*Workplace safety culture*				
Patient safety culture (SAQ)	X	X	X	
Occupational health and safety culture (Modified version of PSC-4)	X	X	X	
Work engagement (COPSOQ)	X	X	X	
*Health*				
General health (COPSOQ)	X	X	X	
Sleep problems (Aronsson et al.)	X	X	X	
Stress (SISQ)	X	X	X	
Health- and work environment-related problems (modified version of Lohela Karlsson et al.)	X	X	X	
Burnout (BAT-4)	X	X	X	
Productivity loss (modified version of Lohela Karlsson et al.)	X	X	X	
Quality of care (RN4CAST)	X	X	X	
Patient safety (RN4CAST)	X	X	X	
***Register data***				
Short-term sick leave (up to 14 days)	X	X	X	X
**Other measures**				
***Self-reported measures***				
Psychological safety (SAQ/SCORE)	X	X	X	
Participated in the education (core component 1)		X		
Participation in in the daily reflections (core component 2)		X	X	

The primary outcomes are assumed to be long term effects of the intervention, whereas the secondary outcomes are assumed to be short-term or intermediate outcomes. Additionally, some other measures will be collected, which are primarily for evaluating potential effects of mediating or moderating factors. Participant characteristics such as sex, age, educational level, occupation, years at current workplace, employment status, working hours, and extent of overtime are collected using self-reported questionnaires in conjunction with the participants answering their first questionnaire.

The health economic evaluation will be conducted to determine the cost-effectiveness and cost-consequence of the intervention in relation to the control condition using primary outcomes. Cost data, including resource use and indirect costs, will be collected through questionnaires and interviews and through documentation related to the different intervention components.

### Primary Outcomes

Intervention effectiveness is measured using two complementary primary outcomes, related to OSH and PS respectively, of which both needs to be improved to assume intervention effectiveness. These outcomes are sick leave and quality of care.

### Sick leave

Sick leave is measured as total number of sick leave days. It is assessed using register data from the healthcare organizations employee registers. Data is collected starting 4 months before intervention start in step one and followed for twelve months after intervention start in step three, see Fig. 3.

### Quality of care

Quality of care is measured as readmission rate within 30-days of discharge. Data is collected starting 4 months before intervention start in step one and followed for twelve months after intervention start in step three. It is assessed from the regions healthcare registers and followed on individual level, clustered to each unit.

### Secondary Outcomes

The short- and intermediate term effects of the intervention are measured with several complementary measures at 4 and 8 months after intervention. Short-term effects are related to working conditions, whereas the intermediate effects are related to HCW well-being and patient outcomes. The short-term outcomes are assumed to improve to achieve the proposed intermediate effects as outlined in Fig. 1.

### Job demands

Job demands are assessed as change in perceived job demands using the Swedish validated version of COPSOQ [43]. It includes the dimensions quantitative demands (three items), work pace (two items) and emotional demands (three items). All items are rated on a 5-point Likert scale ranging from 0 = Always to 100 = Never/Almost never.

### Job resources

Job resources are assessed as changes in perceived job resources. Job resources in this study are measured as local leadership, perception of management, teamwork climate, horizontal trust, meaningful work and influence at work. Local leadership is measured with a Swedish translation of the validated instrument SCORE: Assessment of your work setting, Safety, Communication, Operational Reliability, and Engagement [44] and consists of five items. The items are rated on a 5-point Likert scale ranging from 1 = Disagree strongly to 5 = Agree strongly. The items could also be rated as *Not applicable*. Perception of management, teamwork climate and influence at work are assessed using the validated Swedish version of Safety Attitude Questionnaire (SAQ) [45]. Perception of management is measured with four items. Teamwork climate is measured with six items whereas influence at work is measured using a single item. The items are rated on a 5-point Likert scale ranging from 1 = *Disagree strongly* to 5 = *Agree strongly*. The response option *Not applicable* is also optional. Horizontal trust and meaningful work are both measured with single items questions using the Swedish validated version of COPSOQ [43]. The items are rated on a 5-point Likert scale ranging from 0 = To a very small extent to 100 = To a very large extent.

### Learning environment

Learning environment is a measure that captures the extent to which a climate of learning is established and maintained in a given work setting. It is measured with five items using a Swedish translation of the validated instrument SCORE [44]. Response option ranges from 1 = Disagree strongly to 5 = Agree strongly. The items could also be rated as *Not applicable*.

### Safety climate

Safety climate is measured using both OSH culture as well as PS culture. The outcomes will be evaluated as a change in perceived safety climate, using both perspectives. OSH culture is measured using a revised version of the four item instrument Psychosocial safety climate (PSC-4) [46] where the wording ”psychosocial” or similar has been replaced by the broader concept work environment, which includes both the psychosocial and physical work environment perspectives. Each item is rated on 5-point Likert scale ranging from 1 = Disagree strongly to 5 = Agree strongly. PS culture is measured using the validated Swedish version of Safety Attitude Questionnaire (SAQ) [45]. It comprises seven items, rated on a scale ranging from 1 = *Disagree strongly* to 5 = *Agree strongly*. The response option *Not applicable* is also optional. A new item was developed for the research project capturing management prioritization of OSH in relation to PS. Each item is rated on 5-point Likert scale ranging from 1 = Disagree strongly to 5 = Agree strongly.

### Work environment

To capture a general perception of the work environment a single item measure is used. The question comes from the Swedish version of the RN4CAST-questionnaire used in the European research project Nurse Forecasting: Human Resources Planning in Nursing [47]. The response option ranges from 1 = Poor to 4 = Excellent. The outcome will be evaluated as a change in perception of the work environment.

### Work engagement

Work engagement is assessed using the Swedish version of the validated questionnaire COPSOQ [43]. This measure comprises three items with a response scale ranging from never = 0 to always = 100, i.e., a higher score indicates higher work engagement. The outcome will be measured as change in work engagement.

### Health care worker health

Health is measured as general health, sleep problems, stress, health- and work environment-related problems and burnout. General health is measured using a single item question from the validated Swedish version of COPSOQ [43]. Response options are rated on a 5-point Likert scale ranging from 0 = Poor to 100 = Excellent. Sleep problems is captured through the single item question “Do you have difficulties in sleeping due to thoughts about your work?” [48]. The response options consist of a 6-point Likert scale ranging from 1 = Never to 6 = Every day. Stress is measured using the validated single-item stress question (SISQ), a measure that captures subjective experience of stress and can be seen as a global indicator of stress [49]. Responses are rated on a 5-point Likert scale ranging from 1 = Not at all, to 5 = Very much. Health- and work environment-related problems are measured with a single item question using a revised version of the Swedish validated instrument health-related and work environment-related productivity loss [50–52]. To capture health-related and/or work environment-related problems all HCW will answer to a question on whether they have perceived any health- and/or work environment-related problems while at work the previous month. The response options are yes/no. Burnout is measured using the validated Swedish version of the validated instrument Burnout Assessment Tool (BAT4) [53]. The instrument measures burnout as a syndrome with four core components; exhaustion, mental distance, cognitive and emotional impairment. All items are rated on a five-point scale ranging from 1 = never to 5 = always. The average scores are calculated by adding the scores on all items and then divide this sum by the number of items. The average scale scores vary from 1 to 5 where a lower value indicates less symptoms. All health outcomes will be measured as change in perceived health status of the HCW.

### Productivity loss

To assess change in productivity loss, a revised version of the Swedish validated instrument health-related and work environment-related productivity loss is used [50–52]. HCW responding ”yes” to the question on perceived health-related and/or work environment-related problems while at work the previous month will also answer a follow-up question that captures if the perceived problems have affected their ability to perform at work during the same period. The response options ranges from 0 = Work environment- and/or health-related problems had no effect on my work, to 10 = Work environment- and/or health-related problems completely prevented me from working.

### Short-term sick leave

To measure short-term sick leave the total number of short-term sick leave days (up to 14 days) 12 months after intervention start are assessed. The data is captured from company register data.

### Quality of nursing care

Quality of care is measured as a change in perceived quality of care. A single item question is used, formulated as ”In general, how would you describe the quality of nursing care delivered to patients on your unit/ward?”. The response options ranges on a 4-point Likert scale from 1 = poor to 4 = excellent. The question comes from the Swedish version of the RN4CAST-questionnaire [47] and has been used in previous studies, for example [54].

### Patient safety

Change in perceived PS is followed up using the single item Global patient safety grade, an item that comes from the Swedish version of the RN4CAST-questionnaire [47], previously used in other studies, for example [54]. The item is formulated as “Please give your unit/ward an overall grade on patient safety”. The response options ranges on a 5-point Likert scale from 1 = Failing to 5 = Excellent.

### Other measures

Besides measures of the above presented primary and secondary outcomes additional measures are collected as part of the effectiveness evaluation and used as mediating or moderating factors to assess potential effects of psychological safety and intervention adherence on intervention effectiveness.

Psychological safety is measured using questions from the instruments SAQ and SCORE. It consists of six items, which all are rated on a five-point scale ranging from 1 = Disagree strongly to 5 = Agree strongly. The response option “Not applicable” is also optional in the questions that origin from the SAQ-instrument. The average scores are calculated by adding the scores on all items and then divide this sum by the number of items. It varies from 1 to 5 where a higher value indicates higher psychological safety. The index, which consisted of the same questions has been validated in a previous study [55].

The respondents are also asked whether they took part of the education (yes/no). Those that answer yes are asked how they took part of it. Response options are: Participated physically during a workplace meeting, took part of the digital version of the education, participated in the education provided to mangers and support functions at the beginning of the intervention. They are also asked whether they have participated in the team debriefings during the past four months (yes/no). Those that answered yes are asked to answer how often they did participate. Response options are: Every shift, 2–3 times per week, 2–4 times per month, once per month.

### Dropout and attendance rates

A dropout rate of less than 20% for participating units and an attendance rate of at least 70% of all HCW are assumed in this study. Unit participation in the intervention is primarily assessed through team debriefs, documented using the digital tool designed for documentation and analyzing monthly assessments of work environment and PS issues. Units that fail to report any team debriefs for one month will be contacted by the research team to identify potential reasons for non-compliance and provide guidance to support continued participation. If a unit does not resume conducting and reporting team debriefs in the following month, it will be considered non-adherent to the intervention protocol. However, the unit will remain in the study, and data collection will continue as planned. In the analyses, these units will be handled as non-adherent rather than as dropouts. Reasons for non-adherence will be documented when available. If an individual HCW chooses to withdraw from the study, all data collected up to the point of withdrawal will be included in the analyses. However, no further data will be collected from that individual.

### Statistical methods

Intention-to-treat analyses will be performed for the primary and secondary outcomes. Where relevant, complementary per-protocol analyses will also be conducted. Mixed effects regression models will be used to test for between-group differences in outcomes over time. This model accounts for data nesting, such as HCWs nested within units, by incorporating random effects for clusters and fixed effects for each step [38]. Heterogeneity in intervention effects across clusters will be explored by including interaction terms between clusters and intervention exposure or through stratified analyses.

Since this is a RCT, the risk of confounding can be reduced; however, it may be necessary to adjust for variables such as cluster, number of steps, crossover time, calendar time, and background variables. Possible interaction effects will be assessed. Subgroup analyses may be considered for statistically significant confounders, with adjustments for multiple comparisons where applicable.

Psychological safety, considered an important mechanism of intervention impact, will be analyzed as a potential mediator or moderator. Exploratory analyses will compare units with high adherence to the intervention against those with low adherence, examining potential differences.

Missing data will be addressed through multiple imputations under the assumption of missing at random [56, 57]. Sensitivity analyses will be performed using alternative methods for handling missing data. A detailed statistical analysis plan will be developed in collaboration with a statistician prior to the commencement of data analysis.

### Health economic evaluation

The health economic evaluation will be conducted to determine the cost-effectiveness and cost-consequence of the intervention in relation to the control condition using primary outcomes. The same time range as for the primary outcomes will be used. Results will be presented as incremental cost effectiveness and cost-consequence ratios, i.e., the additional cost for an additional unit of effectiveness. Cost-effectiveness acceptability curves will be applied to capture the statistical uncertainty of the incremental cost-effectiveness ratio. The health economic evaluation will use both an employer and societal perspective as the intervention is assumed to impact both the HCWs and the patients.

Cost data will be collected prospectively from a payer perspective and will include resource use, such as HCW time used for each intervention core component, number of participating HCWs in the different components, consumable materials, administrative costs, etc. Data on resource use will be collected through questionnaires, interviews and through documentation related to the different intervention components. Examples of data include attendance lists, time used for the different meetings, number of reflective meetings. In addition, other potential cost parameters resulting from the intervention will be considered. Indirect costs include productivity loss due to work environment and/or health-related issues, as well as absenteeism. To calculate the value of productivity loss of sick leave from the employer’s perspective, 80% of the daily wage for short-term sick leave (days 1–14), and 10% for long-term sick leave, i.e., between days 15–90, will be used. This is in accordance with the Swedish regulations on employer responsibility. For the analyses from the societal perspective, costs will be based on 100% of the wages for all sick leave days (short-term and long-term) during the follow-up period. Productivity loss due to ill-health and work environment-related problems will be calculated using the number of hours of lost productivity multiplied by hourly wages. Average national salaries and payroll taxes for the reported HCW category will be used to estimate costs for productivity loss and time used in the intervention. All costs will be valued in Swedish kronor and inflated to 2024 year´s values using the consumer price index. An annual discount rate of 3% will be applied for both the costs and the consequences where applicable.

### Process evaluation

In addition to investigating the efficacy and cost-effectiveness of SIOHPS as outlined in this study protocol, the overarching project aims to achieve further objectives that prioritize process evaluation and the implementation of SIOHPS within the MRC framework Guidance [18]. The specifics of these objectives will be delineated in a forthcoming study protocol.

## Discussion

This paper delineates a study protocol for a pragmatic, stepped wedge cluster randomised controlled trial in Swedish healthcare settings. The objective of the trial is to evaluate the SIOPHS method for the systematic integration of OSH and PS management. In addition to the evaluation of the intervention, the project includes embedded economic and process evaluation.

To ensure long-term sustainability in healthcare organisations, actions towards providing good and safe care with high-quality services to patients, and maintaining a healthy workforce, are essential. Such actions, using a more integrated perspective, have been called for, highlighting the potential to achieve synergies and improved results [1, 3]. However, integrated OSH and PS management interventions, developed with the objective of protecting and promoting health and safety of HCW, as well as enhancing PS and quality of care, are currently lacking [6]. Fostering a culture of safety that supports both HCWs and patients might be a promising solution. In response to recent frameworks, such as the Safer Culture Framework [7], interventions are suggested to adopt an integrated approach that targets both enabling and enacting factors. In this project, such an intervention is developed and evaluated guided by the MRC framework [18] using the development approach suggested by O´Cathain and colleagues [20]. The co-development of the programme theory, the intervention and its core components, as well as the evaluation design in collaboration with potential stakeholders and users, is conducted with the objective of strengthening the conditions for the usefulness of the intervention and its effect in the organization. The design of the effectiveness study allows for the assessment of primary and secondary outcomes as well as the cost-effectiveness and cost-consequence of the intervention. Furthermore, an additional study protocol will be published that will extend the scope of this study and provide a more comprehensive examination of the intervention process (implementation, context, and mechanism of impact) in accordance with the MRC framework. If the intervention is found to be effective in the present study context, it will have significant implications for healthcare organisations striving to ensure long-term sustainability and safe care. Additionally, SIOHPS will contribute to the existing theory on safety culture interventions by considering the integration of these areas.

## Supplementary Information

Below is the link to the electronic supplementary material.

Supplementary Material 1

Supplementary Material 2

## Data Availability

Project data sets will be stored at a secure storage for research data provided by Region Västmanland. Members of the SIOPHS project core team will be given access to the cleaned data sets. To ensure confidentiality, all data forms and data files will be pseudonymised and project team members will be blinded of any identifying participant information. The code key will be stored separate. Access to the code key is restricted to researchers in the core research team. The SIOPHS project group will be open for collaboration with other researchers and data will be shared, provided that a research plan is submitted to the project group and aligns with the ethical approval.

## Declarations

### Dissemination

SIOHPS data will be published in peer-reviewed journals and presented at international conferences. Reports of results at an aggregated level will be delivered to, and discussed with, participating workplaces. However, they will not receive information that can be tracked down to individual HCW. Results from the study will be continuously discussed with the extended project group tied to the research project, which consists of researcher and practitioners with expert knowledge of relevance for the project and functions as an advisory board. Also, results will be discussed with previously identified stakeholders involved in the project development phase.

### Trial registration and status

Protocol version 1, 2025–02–17. Recruitment began July 1, 2024, and preliminary date for recruitment completion is December 31, 2025. Any important protocol modification will render an additional application to the Swedish ERA and a modification of the trial registration in ClinicalTrials.gov (Identifier: NCT06398860).

**Table:**

Primary registry and trial identifying number	ClinicalTrials.gov NCT06398860
Date of registration in primary registry	3 May, 2024
Source(s) of monetary or material support	Swedish Research Council for Health, Working Life and Welfare (FORTE) diary number 2022 − 00404. Region Västmanland, internal research funds, diary number LTV-1003601
Primary sponsor	Swedish Research Council for Health, Working Life and Welfare (FORTE)
Secondary sponsor	Region Västmanland
Contact for public queries	Malin Lohela Karlsson, Associate professor, Region Västmanland, Sweden. Phone: 0046214818537, malin.lohela.karlsson@regionvastmanland.se
Contact for scientific queries	Malin Lohela Karlsson, Associate professor, Region Västmanland, Sweden. Phone: 0046214818537, malin.lohela.karlsson@regionvastmanland.se
Public title	A structured method for systematic and integrated occupational safety and health and patient safety management systems (SIOHPS)
Scientific title	Effectiveness- and cost effectiveness of a structured method for systematic and integrated occupational safety and health and patient safety management systems (SIOHPS) – A pragmatic stepped wedge cluster randomised controlled trial
Countries of recruitment	Sweden
Health condition(s) or problem(s) studied	Worker health and quality of care
Intervention(s)	Structured method for systematic and integrated occupational safety and health and patient safety management systems with three core components; (1) targeted education, (2) daily team debriefing and (3) support for systematic management
Comparator	No intervention/TAU
Key inclusion and exclusion criteria	*Inclusion Criteria*: • Individual staff needs to work at least 50% at the workplace • Workplace provides 24-hour care *Exclusion Criteria*: • Plan to implement another method for conduction daily work environment and/or patient safety work during the project period • Units providing pediatric and psychiatric care
Study type	Interventional Allocation: stepped wedge cluster-controlled design with open cohort, where workplace acts as a cluster. Intervention model: Crossover Assignment Masking: Single (Outcomes Assessor) Primary purpose: Health Services Research
Date of first enrolment	July 2024
Target sample size	750
Recruitment status	Enrolling by invitation
Primary outcome(s)	Sick leave (time frame: 12 months) Quality of care (time frame: 12 months)
Key secondary outcomes	Job demands (time frame: 4, 8 months) Job resources (time frame: 4, 8 months) Learning environment (time frame: 4, 8 months) Safety climate (time frame: 4, 8 months) Work environment (time frame: 4, 8 months) Work engagement (time frame: 4, 8 months) Productivity loss (time frame: 4, 8 months) Health (time frame: 4, 8 months) Short-term sick leave (time frame: 12 months) Quality of care (time frame: 4, 8 months) Patient Safety (time frame: 4, 8 months) Incremental cost effectiveness ratios (ICER): Sick leave and Quality of care (time frame: 4 months before first intervention enrollment to 8 months after last intervention enrollment)

## Abbreviations

BAT4: Burnout Assessment Tool 4–item
CONSORT: Consolidated Standards of Reporting Trials
COPSOQ: Copenhagen Psychosocial Questionnaire
HCW: Health care workers
HR: Human resources
JDR: Job Demand Resources theory
MRC: Medical Research Council
OSH: Occupational safety and health
OSH culture: Occupational safety and health culture
PS: Patient safety
PS culture: Patient safety culture
PSC: 4–Psychosocial safety climate questionnaire 4–item
p: SWD–Pragmatic stepped wedge cluster–controlled design
RN4CAST: Register Nurse Forecasting
SAQ: Safety Attitude Questionnaire
SCORE: Assessment of your work setting, Safety, Communication, Operational Reliability, and Engagement
SIOHPS: Systematic and Integrated Occupational safety and Health and Patient safety management Systems
SISQ: singel–item stress question
TIDieR: Template for Intervention Description and Replication
