# Supplementary material for: Effectiveness- and cost effectiveness of a structured method for systematic and integrated occupational safety and health and patient safety management systems (SIOHPS) – a study protocol for a pragmatic stepped wedge cluster randomised controlled trial
Source: BMC Health Serv Res. 2025 Oct 22;25:1391. doi: 10.1186/s12913-025-13537-4 (PMC12541998; doi:10.1186/s12913-025-13537-4)
Supplement: Supplementary file 1 — Supplementary Material 1 [file 12913_2025_13537_MOESM1_ESM.docx]

**Information about participation in the research project "SIOHPS" - a structured method for Systematic and Integrated Occupational safety and Health and Patient Safety management systems**

We would like to ask you if you would like to participate in a research project. In this document, you will receive information about the project and what it means to participate.

**What is the project and why do you want me to participate?**

At the Center for Clinical Research, Region Västmanland, research is conducted on the impact of the work environment on employees' health, performance, and patient safety. This is done in collaboration with researchers from Uppsala University, Karolinska Institutet, and the University of Gävle. The aim of the research is to contribute to a good work environment and good and safe care. An important part is to evaluate interventions that have good potential to work in healthcare organisations.

Research shows clear associations between occupational health and safety as well as patient safety, and the areas are described as "two sides of the same coin." Traditionally, these areas have been handled in separate regulations and routines, but in recent years, there has been a request to work with these areas more integratively. SIOHPS is a method developed to work integratively with occupational health and safety as well as patient safety in everyday life. The method follows existing laws and regulations while being adapted to the conditions of healthcare. In addition to identifying risks and adverse events, it also provides support in strengthening what works well in the workgroup. SIOHPS is based on employee participation, communication, and learning, which are important prerequisites for creating a good work environment and safe care.

The aim of the project is to investigate whether the SIOHPS method can contribute to improved work environment and health among healthcare workers while also enhancing patient safety and quality of care. A digital tool supports the process by visualize the work, providing methodological guidance, facilitating administration, and simplifying monitoring for managers, patient safety representatives and safety representatives.

You are invited to participate in the study because you work at a workplace where the SIOHPS method is planned to be implemented. Your contact details were provided by your immediate supervisor.

**How is the study conducted?**

All workplaces will implement the SIOHPS method but will start at different times. You will receive information and training on the method during a workplace meeting before implementation, where you will also have the opportunity to ask questions.

If you choose to participate in the study, you will be asked to complete a questionnaire via your work email before the start and again after 4, and 8 months. The questionnaire covers topics such as working conditions, health, performance, quality of nursing care, patient safety, safety culture, and psychological safety. It takes approximately 15 minutes to complete. These self-assessments are essential for evaluating the effects of the intervention.

An important part of the study is to examine whether the implementation of the method can contribute to reduced sick leave. To evaluate this, information needs to be collected from the region’s personnel system.We will also monitor patient safety in the workplace by gathering data on indicators such as pressure ulcer prevalence, unplanned readmissions, and 30-day mortality.

Additionally, a member of the research team will participate as an observer when you and your collegaues conduct the method at your workplace on two different occasions. You will receive verbal information beforehand and will have the option to decline participation in the session.

If you wish to participate in the study, you consent by filling out the informed consent form before starting to fill in the questionnaire. You can choose to consent to both completing the questionnaire and allowing the collection of register data on sick leave, or you can consent to only completing the questionnaire or only allowing the collection of register data on sick leave.

**Possible consequences and risks of participating in the study**

Participation in the study is not expected to involve any risks. If you experience any negative consequences during or after the study, you can contact the principal investigator of the project.

**What happens to my data?**

The project will collect and register your responses to the questionnaires you have completed. Your personal data will be replaced with an identification number, allowing us to link your responses over time. The data will then be assigned a unique code, ensuring that any information directly traceable to an individual is removed. When the study results are presented, it will not be possible to see what you have answered. No information that could identify your answers will be available to managers or the respective region as your employer.

A code key, which allows the responsible researcher to identify an individual participant in exceptional cases, will be securely stored separately from the collected data. All data will be securely stored in accordance with Region Västmanland’s guidelines for research data storage and retained for 10 years to enable review.

Your responses and results will be handled so that unauthorized persons cannot access them. The responsible party for your personal data is Region Västmanland, www.regionvastmanland.se, 021-17 30 00. Under the EU General Data Protection Regulation (GDPR), you have the right to access the data collected about you in the study free of charge and to request corrections if needed. You may also request the deletion of your data or the restriction of its processing. However, the right to deletion and restriction of processing of personal data does not apply when the data is necessary for the ongoing research. If you wish to access your data, please contact the principal investigator, Malin Lohela Karlsson. The Data Protection Officer can be reached at [dataskyddsombudet@regionvastmanland.se](mailto:dataskyddsombudet@regionvastmanland.se), tel.nr: 021-17 30 00. If you are dissatisfied with how your personal data is being processed, you have the right to file a complaint with the Swedish Authority for Privacy Protection (IMY), which is the supervisory authority.

**How do I get information about the results of the study?**

All participating workplaces will receive feedback on the workplace's result at a workplace meeting after the final measurement have been completed. The results of the entire study will then be compiled into research reports and presented in scientific articles.

**Insurance and compensation**

You are insured as an employee through your employment. No compensation is provided for participation. Gift cards worth 200 SEK, which can be used at several places, will be raffled among those who complete the questionnaires during the study.

**Participation is voluntary**

Your participation is voluntary, and you can choose to withdraw at any time. If you choose not to participate or wish to withdraw your participation, you do not need to provide a reason. It will not be reported to your manager who has chosen to withdraw from the study.

If you wish to withdraw your participation, you should contact the project team (see below).

**Project Team SIOPHS:**

**Principal investigator:** Malin Lohela Karlsson, Associate professor, Senior Analyst, Health Economist, Region Västmanland, Center for Clinical Research, Region Västmanland/Uppsala University

Email: [malin.lohela.karlsson@regionvastmanland.se](mailto:malin.lohela.karlsson@regionvastmanland.se)

Phone: 021-481 85 37 

**Project Leader Västmanland:** Ann-Sofie Ersson, Specialist Psychologist in Work and Organizational Psychology, PhD Candidate, Region Västmanland, Center for Clinical Research, Region Västmanland/Uppsala University

E-mail: [ann-sofie.ersson@regionvastmanland.se](mailto:ann-sofie.ersson@regionvastmanland.se)

Phone: 021-173583

**Project Leader Dalarna:** Camilla Göras, PhD, specialist registered nurse, Lecturer, Faculty of Health and Occupational Sciences, Department of Caring Sciences, University of Gävle

Email: [camilla.goras@hig.se](mailto:camilla.goras@hig.se)

Phone: 026 - 648468

**Consent to participate in the SamSa study**

I have received oral and written information about the study and have had the opportunity to ask questions. I will keep the written information. By filling in the information below, I consent to my data being processed as described in the information regarding participation in the SIOHPS study under the section "What happens to my data?".

I consent to participate in the SIOHPS study and will receive questionnaires sent to my work email.

I consent to the collection of sick leave statistics from the employer's personnel system for use in the research study.

| **Place and date** | **Name** | **Signature** |
| --- | --- | --- |
|  |  |  |
